# Supplementary material for: Chemokine (C-C Motif) Ligand 8 and Tubulo-Interstitial Injury in Chronic Kidney Disease
Source: Cells. 2022 Feb 14;11(4):658. doi: 10.3390/cells11040658 (PMC8869891; doi:10.3390/cells11040658)
Supplement: Supplementary file 1 [file cells-11-00658-s001.zip › cells-1471891-supplementary.pdf]

Table S1. Primer sets used for qRT-PCR

Genes	Forward (5'->3')	Reverse (5'->3')
IL-8	ATGACTTCCAAGCTGGCCGTGGCT	TCTCAGCCCTCTTCAAAACTTCTC
P53	CCCCTCCTGGCCCCTGTCATCTTC	GCAGCGCCTCACAACCTCCGTCAT
CCR8	GCTGTGTGAACCCTGTTATCT	CTCTCCCTAGGCATTTGTCTTC
CCR2	CTGGAAGGTGTTCAGGAGAATG	CGAAACGAGAAGAAGAGGCATAG
FN	CCACCCCCATAAGGCATAGG	GTAGGGGTCAAAGCACGAGTCATC
GAPDH	TCGACAGTCAGCCGCATCT	CCGTTGACTCCGACCTTCA

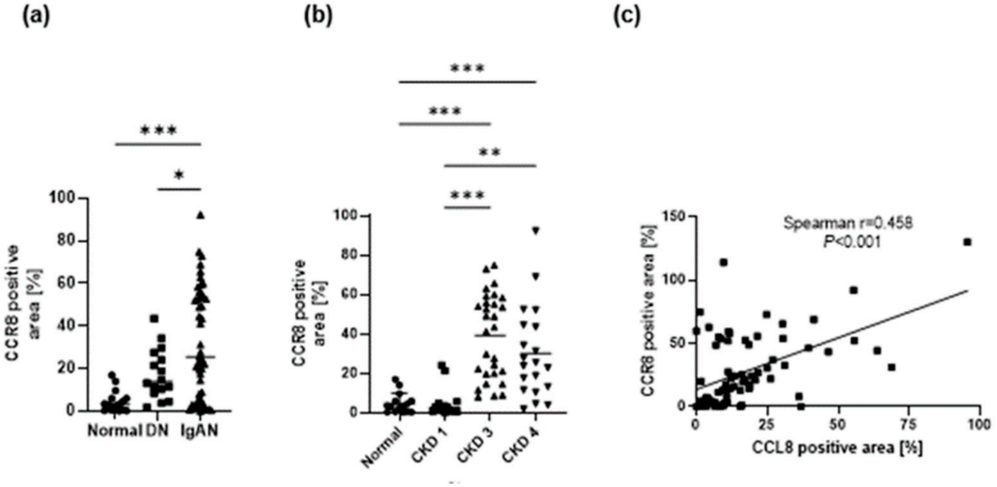

Figure S1. The expression of CCR8 in chronic kidney disease. (**a**,**b**) CCR8 expression levels were significantly higher in patients with IgA nephropathy and CKD stages 3 and 4. (**c**)The percentage of CCR8 positively correlated with that of the CCL8. *$p < 0.05$, ** $p < 0.01$, and *** $p < 0.001$
